# Supplementary material for: Enhanced Antitumor Efficacy of Cytarabine and Idarubicin in Acute Myeloid Leukemia Using Liposomal Formulation: In Vitro and In Vivo Studies
Source: Pharmaceutics. 2024 Sep 19;16(9):1220. doi: 10.3390/pharmaceutics16091220 (PMC11434936; doi:10.3390/pharmaceutics16091220)
Supplement: Supplementary file 1 [file pharmaceutics-16-01220-s001.zip › pharmaceutics-3182053-supplementary.pdf]

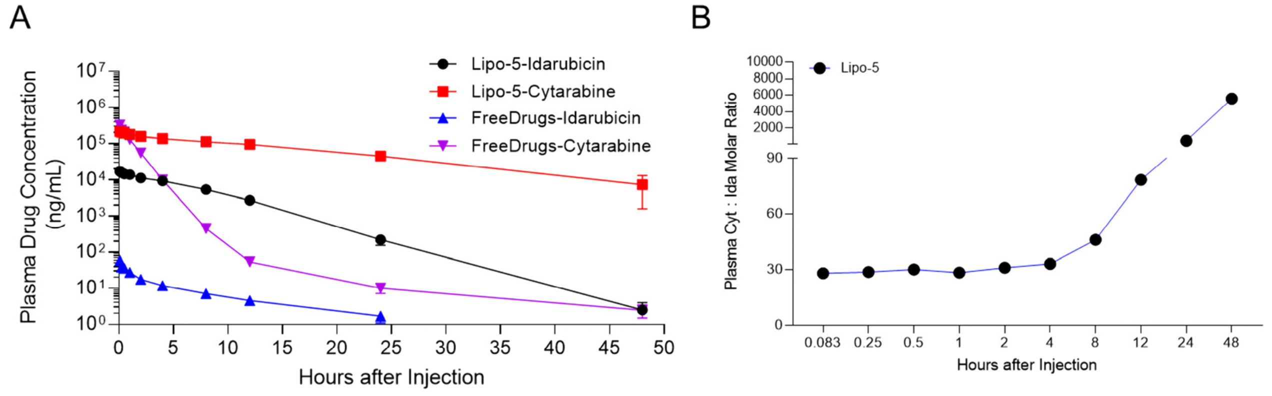

Figure S1: PK characteristics of SD rats treated with cytarabine-idarubicin liposomes. (A)Plasma drug concentration time curve. (B)Plasma cytarabine/idarubicin molar ratio time curve.
